# Vigilância epidemiológica da transmissão vertical da sífilis: dados de seis unidades federativas no Brasil

**DOI:** 10.26633/RPSP.2017.44

**Published:** 2017-04-14

**Authors:** Valeria Saraceni, Gerson Fernando Mendes Pereira, Mariangela Freitas da Silveira, Maria Alix Leite Araujo, Angelica Espinosa Miranda

**Affiliations:** 1 Secretaria Municipal de Saúde do Rio de Janeiro Superintendência de Vigilância em Saúde Rio de Janeiro (RJ) Brasil Secretaria Municipal de Saúde do Rio de Janeiro, Superintendência de Vigilância em Saúde, Rio de Janeiro (RJ), Brasil.; 2 Ministério da Saúde, Secretaria de Vigilância em Saúde Departamento de DST/AIDS Brasília (DF) Brasil Ministério da Saúde, Secretaria de Vigilância em Saúde, Departamento de DST/AIDS, Brasília (DF), Brasil.; 3 Universidade Federal de Pelotas Departamento Materno-Infantil Pelotas (RS) Brasil Universidade Federal de Pelotas, Departamento Materno-Infantil, Pelotas (RS), Brasil.; 4 Universidade de Fortaleza Universidade de Fortaleza Fortaleza (CE) Brasil Universidade de Fortaleza, Programa de Pós-Graduação em Saúde Coletiva, Fortaleza (CE), Brasil.; 5 Universidade Federal do Espírito Santo Universidade Federal do Espírito Santo Vitória (ES) Brasil Universidade Federal do Espírito Santo, Programa de Pós-Graduação em Saúde Coletiva, Vitória (ES), Brasil.

**Keywords:** Sífilis, gestação, sífilis congênita, profissionais da saúde, vigilância sanitária, Brasil, Pregnancy, syphilis, congenital, health personnel, health surveillance, Brazil, Embarazo, sífilis congénita, personal de salud, vigilancia sanitaria, Brasil

## Abstract

**Objetivo.:**

Descrever o perfil epidemiológico dos casos notificados de sífilis em gestantes e sífilis congênita nos estados brasileiros do Amazonas, Ceará, Espírito Santo, Rio de Janeiro e Rio Grande do Sul e no Distrito Federal a partir de dados do Sistema Nacional de Agravos de Notificação (SINAN).

**Métodos.:**

*Estudo descritivo incluindo avaliação ecológica e transversal. Foram utilizados dados do SINAN Net. Foram calculadas a taxa de detecção de sífilis em gestantes e a taxa de incidência de sífilis congênita por 1 000 nascidos vivos. Para identificar as gestantes notificadas com sífilis com desfecho de sífilis congênita, as duas bases do SINAN foram relacionadas por meio do* software *RecLink. Como os dados eram de representatividade regional, as comparações foram feitas entre as unidades da federação, e não com a soma dos casos.*

**Resultados.:**

A taxa de detecção de sífilis em gestantes cresceu entre 21% (Amazonas) e 75% (Rio de Janeiro). A incidência de sífilis congênita seguiu o mesmo perfil de incremento, variando de 35,6% no Distrito Federal a 639,9% no Rio Grande do Sul, com redução de 0,7% no Amazonas. A realização de pré-natal nas mulheres com desfecho de sífilis congênita variou de 67,3% no Amazonas a 83,3% no Distrito Federal. Das gestantes com sífilis, 43% tiveram desfecho notificado de sífilis congênita. Nas gestantes com sífilis e desfecho de sífilis congênita, o diagnóstico materno ocorreu durante o pré-natal em 74% e no parto em 18%. Em 8% das mulheres ignorava-se o momento do diagnóstico.

**Conclusão.:**

O incremento nas taxas de detecção de sífilis pode ter resultado do aumento na notificação. O monitoramento constante em gestantes é essencial para a eliminação desses agravos.

## Abstract (en)

***Objective*.:**

To describe the epidemiological profile of reported cases of syphilis in pregnancy and congenital syphilis in five states (Amazonas, Ceará, Espírito Santo, Rio de Janeiro, and Rio Grande do Sul) and the Federal District using data from the Reportable Disease Information System (SINAN).

***Method*.:**

This descriptive study including an ecological and cross-sectional evaluation employed data from SINAN Net. The syphilis detection rate in pregnancy and the congenital syphilis incidence rate per 1 000 live births were calculated. To identify pregnant women with syphilis who had an outcome of congenital syphilis, the two SINAN databases were linked using the RecLink software. Because the data were representative at the state (not national) level, comparisons were made between the units of the federation and not with the sum of cases.

***Results*.:**

A growth in the syphilis detection rate in pregnancy was detected, ranging from 21% (Amazonas) to 75% (Rio de Janeiro) during the study period. The incidence of congenital syphilis followed the same trend of growth (ranging from 35.6% in the Federal District to 63.9% in Rio Grande do Sul), except for a 0.7% decline in Amazonas. The proportion of women with an outcome of congenital syphilis who had prenatal care ranged from 67.3% in Amazonas to 83.3% in the Federal District. Of the pregnant women with syphilis, 43% had an outcome of congenital syphilis. In pregnant women with syphilis and an outcome of congenital syphilis, maternal diagnosis was made prenatally in 74% and at delivery in 18%. The moment of diagnosis was ignored in 8% of the women.

***Conclusion*.:**

The increase in the syphilis detection rate may have resulted from an increase in the report rate. Ongoing monitoring of pregnant women is essential to eliminate syphilis.

## RESUMEN

Describir el perfil epidemiológico de los casos notificados de sífilis en embarazadas y de sífilis congénita en los estados brasileños de Amazonas, Ceará, Espírito Santo, Rio de Janeiro y Rio Grande do Sul y en el Distrito Federal a partir de datos del Sistema Nacional de Información de Enfermedades de Notificación Obligatoria (SINAN).

Estudio descriptivo que incluye una valoración ecológica y transversal. Se utilizaron datos de la Red SINAN. Se calcularon la tasa de detección de sífilis en embarazadas y la tasa de incidencia de sífilis congénita por 1000 nacidos vivos. Para identificar a las embarazadas notificadas con sífilis que causó sífilis congénita, se relacionaron las dos bases del SINAN por medio del software RecLink. Como los datos eran representativos a nivel regional, las comparaciones se hicieron entre las unidades de la federación y no con la suma de los casos.

La tasa de detección de sífilis en embarazadas aumentó entre 21% (Amazonas) y 75% (Rio de Janeiro). La incidencia de sífilis congénita siguió el mismo perfil de incremento, variando de 35,6% en el Distrito Federal a 63,9% en Rio Grande do Sul, con una reducción de 0,7% en Amazonas. Los controles prenatales en las mujeres que tuvieron hijos con sífilis congénita variaron de 67,3% en Amazonas a 83,3% en el Distrito Federal. De las embarazadas con sífilis, en el 43% de los casos se notificó sífilis congénita. En las embarazadas con sífilis que causó sífilis congénita, el diagnóstico materno fue durante la etapa prenatal en 74% de los casos y en el parto en 18% de los casos. En 8% de las mujeres se desconocía el momento del diagnóstico.

***Conclusión*.:**

El incremento de las tasas de detección de sífilis puede haber sido consecuencia de una mayor notificación. Es esencial el seguimiento constante de las mujeres embarazadas para eliminar el aumento de esas enfermedades.

A sífilis, causada pelo *Treponema pallidum*, é uma doença sexualmente transmissível. Quando não tratada durante a gestação, resulta em considerável proporção de mortes fetais e neonatais precoces (1, 2), com alta probabilidade de transmissão vertical, principalmente nas fases primária e secundária, aumentando o risco de mortes perinatais (3).

O último levantamento sobre sífilis entre parturientes no Brasil incluiu uma amostra de aproximadamente 36 000 gestantes, distribuídas entre as cinco regiões geográficas brasileiras (4). A prevalência estimada de sífilis em gestantes foi de 0,85% para o Brasil como um todo, 1,05% para a região Norte, 1,14% para o Nordeste, 0,73% para o Sudeste, 0,48% para o Sul e 1,20% para o Centro-Oeste. Em 2013, observou-se uma taxa de detecção de 7,4 casos de sífilis em gestantes para cada 1 000 nascidos vivos (4).

A sífilis pode ser classificada pelo tempo de infecção como sífilis adquirida recente ou sífilis adquirida tardia; ou pela presença de manifestações clínicas como sífilis primária, secundária, latente, terciária e neurossífilis (1). A transmissão vertical da sífilis, que pode ocorrer em até 25% dos casos (5, 6), pode ser evitada desde que o diagnóstico e o tratamento adequados da gestante sejam realizados durante o período gestacional (5). A sífilis congênita e a sífilis em gestantes são de notificação compulsória no Brasil desde 1986 (7) e 2005 (8), respectivamente, existindo uma definição de caso própria para fins de vigilância epidemiológica, revisada periodicamente, de cada uma dessas situações (9). Portanto, as informações sobre abortos, natimortos e nascidos vivos com sífilis congênita devem ser inseridas no Sistema de Informação de Agravos de Notificação (SINAN) (4, 7). O monitoramento dessas infecções por meio do SINAN é de fundamental importância para a eliminação da sífilis congênita, pois fornece subsídios para o planejamento e a definição das intervenções necessárias (4, 6).

O Ministério da Saúde recomenda a triagem sorológica para sífilis, de preferência por meio do teste rápido treponêmico, no primeiro e terceiro trimestres de gestação e na ocasião da internação para o parto ou curetagem. Para as gestantes com resultado reagente, o controle do tratamento e da cura deve ser realizado através do *Venereal Disease Research Laboratory test* (VDRL), um exame não treponêmico (4–6).

Entretanto, apesar da maior oferta de testes diagnósticos para as gestantes e seus parceiros com a introdução dos testes rápidos distribuídos pelo Ministério da Saúde, a transmissão vertical da sífilis não tem declinado da forma esperada, o que evidencia que somente o acesso ao diagnóstico não é suficiente para garantir a melhoria da qualidade da atenção à gestante portadora de sífilis. O cuidado dispensado à gestante pela Estratégia Saúde da Família, que atualmente é a base da atenção primária à saúde no Brasil, precisa aliar a tecnologia existente à assistência de qualidade para a redução da sífilis congênita (9). Os dados de notificação, quando usados adequadamente, podem contribuir para esse esforço.

Diante desse contexto, o objetivo deste estudo foi descrever o perfil epidemiológico dos casos notificados de sífilis em gestantes e de sífilis congênita nos estados brasileiros do Amazonas, Ceará, Espírito Santo, Rio de Janeiro e Rio Grande do Sul e no Distrito Federal.

## MATERIAIS E MÉTODOS

O presente estudo descritivo dos casos de sífilis em gestantes e de sífilis congênita, incluindo avaliação ecológica e transversal, foi conduzido nos estados do Amazonas, Ceará, Espírito Santo, Rio de Janeiro e Rio Grande do Sul e no Distrito Federal, Brasil. Os dados incluídos tinham representatividade regional. A escolha de estados representando as diferentes regiões geográficas do Brasil foi necessária para contemplar a heterogeneidade no acesso aos serviços de saúde. Os dados utilizados para a análise provieram das bases de dados do SINAN Net para sífilis em gestantes e sífilis congênita. As variáveis incluídas foram as que constam de maneira padronizada nas fichas de investigação dos dois agravos, disponíveis online (http://portalsinan.saude.gov.br/sifilis-em-gestante e http://portalsinan.saude.gov.br/sifilis-congenita), e digitadas no SINAN em cada município de notificação. Todas as fichas de notificação disponíveis no SINAN no período de 2007 a 2012 foram incluídas na análise. Esse intervalo representa o período entre o início da vigência do SINAN versão Net (2007) até o ano mais completo imediatamente antes do início deste projeto.

Definiu-se “município silencioso” como aquele que não apresentou notificação de sífilis em gestantes ou sífilis congênita no período; “tratamento adequado” para sífilis na gestação foi aquele prescrito de acordo com a fase clínica da sífilis e com uso de penicilina benzatina (10); e “exame de líquor positivo” foi considerado aquele que apresentou alterações citológicas, bioquímicas ou VDRL reagente.

As distribuições proporcionais das variáveis sociodemográficas e clínicas disponíveis no SINAN foram comparadas pelo teste do qui-quadrado (χ^2^) de Pearson para homogeneidade de proporções. As medianas de idade foram comparadas por análise de variância (ANOVA). Os dados foram analisados no pacote estatístico Stata 11.2, com significância estabelecida em 5%. Como os dados eram de representatividade regional, as comparações foram feitas entre as seis unidades da federação, e não com a soma dos casos das seis unidades da federação. A taxa de detecção de sífilis em gestantes foi calculada utilizando-se o número de notificações de sífilis em gestantes sobre o número de nascidos vivos no mesmo ano. A taxa de incidência de sífilis congênita foi calculada como o número de notificações sobre os nascidos vivos por 1 000 nascidos vivos no mesmo ano, conforme definição dos indicadores pelo Ministério da Saúde (4). Os dados para esses cálculos são obtidos do Sistema de Informação Sobre Nascidos Vivos (SINASC). Para identificar as gestantes notificadas com sífilis e que também tiveram desfecho de sífilis congênita, foi realizado um relacionamento das duas bases de dados do SINAN por meio do *software* RecLink (11).

O projeto foi aprovado no Comitê de Ética em Pesquisa da Universidade Federal do Espírito Santo (no. 640.580/2014). A privacidade e a confidencialidade foram asseguradas em todas as fases do projeto por meio de codificação. Os bancos de dados foram analisados sem a identificação dos sujeitos.

## RESULTADOS

### Base de dados: sífilis em gestantes

A taxa de detecção de sífilis na gestação por 1 000 nascidos vivos apresentou incremento nos cinco estados estudados entre 2007 e 2012, variando de 21,2% no Amazonas a 75,4% no Rio de Janeiro. Para o Distrito Federal, observou-se redução de 5,2%. O percentual de municípios silenciosos foi de 8,1% no Amazonas, 9,8% no Ceará, 20,5% no Espírito Santo, 13,0% no Rio de Janeiro e 56,7% no Rio Grande do Sul.

A tabela 1 apresenta a distribuição proporcional das características sociodemográficas e clínicas dos casos de sífilis em gestantes. A mediana de idade variou de 23 anos no Amazonas, Ceará e Rio de Janeiro a 27 anos no Distrito Federal. A escolaridade foi maior no Distrito Federal e no Rio Grande do Sul. A heterogeneidade na distribuição de raça/cor acompanhou as características regionais, com maior percentual de indígenas no Amazonas. Esse estado, junto com o Rio de Janeiro, teve maior proporção de casos em residentes na capital, enquanto que o Ceará, o Espírito Santo e o Rio Grande do Sul tiveram mais casos entre residentes em outros municípios. A maior parte das gestantes foi reagente ao teste não treponêmico. É importante mencionar que a utilização de testes treponêmicos apresentou um incremento no período; esse incremento variou de 32,3% no Amazonas a 80,6% no Rio de Janeiro. No Distrito Federal houve uma redução de 25% no uso de testes treponêmicos.

A classificação clínica do estágio da doença variou bastante, com 62,8% dos casos classificados como sífilis primária no Amazonas e 54,7% classificados como ignorados no Rio de Janeiro. O tratamento prescrito para a gestante, que deveria seguir a classificação clínica, apresentou comportamento peculiar. A proporção de casos com tratamento considerado adequado conforme a fase clínica informada variou de 45,5% no Rio de Janeiro a 61,9% no Distrito Federal.

**TABELA 1.** Características sociodemográficas, clínicas e de tratamento dos casos de sífilis na gestação notificados em seis unidades federativas no Brasil, 2007 a 2012

Característica n (%)^a^	Unidade federativa						
	Amazonas No. = 1 533	Ceará No. = 2 764	Distrito Federal No. = 565	Espírito Santo No. = 1 557	Rio de Janeiro No. = 8 728	Rio Grande do Sul No. = 3 163	*P*-valor^b^
Idade (anos), mediana (IQR)	23 (19 a 28)	23 (19 a 28)	27 (22 a 32)	24 (20 a 29)	23 (19 a 28)	25 (20 a 30)	< 0,001
Escolaridade (anos)							
< 8	921 (60,1)	1 503 (54,4)	223 (39,5)	688 (49,2)	3 156 (36,2)	1 748 (55,3)	< 0,001
≥ 9	344 (22,4)	431 (15,6)	131 (23,2)	337 (21,6)	1 266 (14,5)	562 (17,7)	
Ignorado	268 (17,5)	830 (30,0)	211 (37,3)	532 (34,2)	4 306 (49,3)	853 (27,0)	
Raça/cor							
Branca	152 (9,9)	370 (13,4)	153 (27,1)	296 (19,0)	1 644 (18,8)	2 001 (63,3)	< 0,001
Preta	91 (5,9)	234 (8,5)	63 (11,2)	227 (14,6)	1 644 (18,8)	464 (14,7)	
Amarela	26 (1,7)	44 (1,6)	12 (2,1)	13 (0,8)	55 (0,6)	13 (0,4)	
Parda	1 175 (76,7)	1 881 (68,0)	240 (42,5)	830 (53,3)	3 137 (35,9)	379 (12,0)	
Indígena	50 (3,3)	25 (0,9)	0 (0,0)	3 (0,2)	14 (0,2)	39 (1,2)	
Ignorado	39 (2,5)	210 (7,6)	97 (17,1)	188 (12,1)	2 234 (25,5)	267 (8,4)	
Local de residência							
Capital	1 037 (67,7)	752 (27,2)	565 (100,0)	319 (20,5)	5 612 (64,3)	860 (27,2)	< 0,001
Interior	496 (32,3)	2 012 (72,8)	0 (0,0)	1 238 (79,5)	3 116 (35,7)	2 303 (72,8)	
Resultado VDRL							
Reagente	1 263 (82,7)	2 454 (90,4)	506 (89,6)	1 474 (94,7)	8 108 (92,9)	2 963 (3,9)	< 0,001
Não reagente	84 (5,5)	73 (2,7)	8 (1,4)	24 (1,5)	115 (1,3)	52 (1,7)	
Não realizado/ignorado	180 (11,8)	186 (6,9)	51 (9,0)	59 (3,8)	505 (5,8)	140 (4,4)	
Resultado treponêmico							
Reagente	414 (27,1)	717 (26,4)	315 (55,8)	426 (27,4)	2 584 (29,6)	1 151 (36,5)	< 0,001
Não reagente	67 (4,4)	129 (4,8)	29 (5,1)	60 (3,8)	178 (2,0)	146 (4,6)	
Não realizado/ignorado	1 046 (68,5)	1 867 (68,8)	221 (39,1)	1 071 (68,8)	5 966 (68,4)	1 858 (58,9)	
Classificaçao clínica							
Primária	893 (62,8)	1 110 (45,4)	146 (30,4)	362 (26,9)	2 146 (26,9)	1 133 (38,6)	< 0,001
Secundária	110 (7,7)	172 (7,1)	22 (4,6)	125 (9,3)	347 (4,3)	207 (7,1)	
Terciária	43 (3,0)	441 (18,0)	116 (24,1)	158 (11,7)	420 (5,2)	152 (5,2)	
Latente	190 (13,4)	367 (15,0)	55 (11,5)	244 (18,2)	696 (8,7)	276 (9,4)	
Ignorada	187 (13,1)	355 (14,5)	141 (29,4)	455 (33,9)	4 366 (54,7)	1 167 (39,7)	
Tratamento prescrito							
Penicilina benzatina 1 200 000 UI	974 (63,8)	885 (32,6)	60 (10,6)	265 (17,0)	1 566 (17,9)	956 (30,3	< 0,001
Penicilina benzatina 2 400 000 UI	153 (10,0)	196 (7,2)	14 (2,5)	61 (3,9)	363 (4,2)	275 8,7)	
Penicilina benzatina 7 200 000 UI	181 (11,9)	1 295 (47,7)	433 (76,6)	986 (63,3)	4 307 (49,4)	1 329 (42,1)	
Outro	37 (2,4)	58 (2,2)	8 (1,4)	42 (2,8)	168 (1,9)	151 (4,8)	
Não realizado	118 (7,7)	117 (4,3)	14 (2,5)	112 (7,2)	1 352 (15,5)	204 (6,5)	
Ignorado	64 (4,2)	162 (6,0)	36 (6,4)	91 (5,8)	972 (11,1)	240 (7,6)	
Tratamento adequado^c^							
Não	728 (47,5)	1 282 (46,4)	215 (38,1)	739 (47,5)	4 751 (54,4)	1 611 (50,9)	< 0,001
Sim	805 (52,5)	1 482 (53,6)	350 (61,9)	818 (52,5)	3 977 (45,5)	1 552 (49,1)	

***Fonte:*** SINAN.

a Exceto idade.

b Teste de homogeneidade de proporções pelo qui-quadrado de Pearson.

c Tratamento adequado para a fase clinica da doença de acordo com o Manual do Ministério da Saúde (10).

### Base de dados: sífilis congênita

A taxa de incidência de sífilis congênita por 1 000 nascidos vivos cresceu em quatro estados e no Distrito Federal entre 2007 e 2012; o incremento variou de 35,6% no Distrito Federal a 63,9% no Rio Grande do Sul. No Amazonas, foi registrada uma redução de 0,7% na taxa de sífilis congênita no período. O percentual de municípios silenciosos foi de 45,2% no Amazonas, 24,5% no Ceará, 34,6% no Espírito Santo, 28,3% no Rio de Janeiro e 68,8% no Rio Grande do Sul.

A tabela 2 apresenta a distribuição proporcional das características sociodemográficas e clínicas das mulheres com desfecho notificado de sífilis congênita. A mediana de idade variou de 23 anos no Amazonas, Ceará e Rio de Janeiro a 26 anos no Distrito Federal. As mulheres do Distrito Federal, Espírito Santo e Rio de Janeiro apresentaram maior escolaridade quando comparadas às mulheres de outros estados. A heterogeneidade na distribuição de raça/cor acompanhou as características regionais, com maior percentual de indígenas no Amazonas.

**TABELA 2.** Características sociodemográficas, clínicas e de tratamento das mulheres com desfecho notificado de sífilis congênita em seis unidades federativas no Brasil, 2007 a 2012

Característica n (%)^a^	Unidade federativa						
	Amazonas No. = 869	Ceará No. = 4 045	Distrito Federal No. = 545	Espírito Santo No. = 949	Rio de Janeiro No. = 9 820	Rio Grande do Sul No. = 2 745	*P*-valor^b^
Idade (anos), mediana (IQR)	23 (20 a 28)	23 (19 a 28)	26 (22 a 31)	24 (20 a 29)	23 (19 a 28)	25 (21 a 31)	< 0,001
Escolaridade (anos)							
< 8	556 (64,0)	2 520 (62,3)	273 (50,1)	443 (46,7)	4 540 (46,2)	1 743 (63,5)	< 0,001
≥ 9	202 (23,2)	685 (16,9)	124 (22,7)	179 (18,8)	1 489 (15,2)	414 (15,1)	
Ignorado	111 (12,8)	840 (20,8)	148 (27,2)	327 (34,5)	3 791 (38,6)	588 (21,4)	
Raça/cor							
Branca	83 (9,6)	264 (6,5)	101 (18,5)	136 (14,3)	1 656 (16,9)	1 684 (61,4)	< 0,001
Preta	67 (7,7)13	124 (3,1)	26 (4,8)	117 (12,3)	1 967 (20,0)	553 (20,1)	
Amarela	9 (1,0)	20 (0,5)	4 (0,7)	4 (0,4)	30 (0,3)	5 (0,2)	
Parda	671 (77,2)	3 345 (82,7)	328 (60,2)	563 (59,3)	4 138 (42,1)	312 (11,4)	
Indígena	16 (1,8)	14 (0,3)	0 (0,0)	1 (0,1)	8 (0,1)	6 (0,2)	
Ignorado	23 (2,7)	278 (6,9)	86 (15,8)	128 (13,5)	2 012 (20,6)	185 (6,7)	
Local de residência							
Capital	726 (83,5)	2 684 (66,4)	545 (100,0)	141 (14,9)	6 185 (63,0)	1 213 (44,2)	< 0,001
Interior	143 (16,5)	1 316 (33,6)	0 (0,0)	808 (85,1)	3 635 (37,0)	1 532 (55,8)	
Realizaêão pré-natal							
Sim	585 (67,3)	2 836 (70,1)	454 (83,3)	713 (75,1)	6 667 (67,9)	2 072 (75,5)	< 0,001
Não	266 (30,6)	1 014 (25,1)	84 (15,4)	202 (21,3)	1 865 (19,0)	597 (21,8)	
Ignorado	18 (2,1)	195 (4,8)	7 (1,3)	34 (3,6)	1 288 (13,1)	76 (2,7)	
Diagnóstico de sífilis							
Pré-natal	274 (31,5)	1 616 (40,0)	281 (51,6)	472 (49,7)	3 511 (35,8)	1 403 (51,1)	< 0,001
Parto	519 (59,7)	2 157 (53,3)	212 (38,9)	403 (42,5)	5 120 (52,1)	1 128 (41,1)	
Pós-parto	36 (4,2)	91 (2,2)	9 (1,6)	31 (3,3)	204 (2,1)	39 (6,4)	
Não realizado/ignorado	40 (4,6)	181 (4,5)	43 (7,9)	43 (4,5)	985 (10,0)	175 (1,4)	
VDRL no parto							
Reagente	820 (94,5)	3 798 (93,9)	507 (93,2)	860 (90,6)	9 346 (95,2)	2 568 (93,6)	< 0,001
Não reagente	11 (1,3)	60 (1,5)	17 (3,1)	38 (4,0)	164 (1,7)	61 (2,2)	
Não realizado/ignorado	37 (4,2)	187 (4,6)	20 (3,7)	51 (5,4)	308 (3,1)	116 (4,2)	
Teste treponemico no parto							
Reagente	218 (25,1)	781 (19,3)	241 (44,2)	127 (13,4)	2 879 (29,3)	1 112 (40,5)	< 0,001
Não reagente	29 (3,3)	118 (2,9)	21 93,9)	30 (3,1)	338 (3,4)	94 (3,4)	
Não realizado/ignorado	622 (71,6)	3 146 (77,8)	283 (51,9)	792 (83,5)	6 603 (67,3)	1 539 (56,1)	
Tratamento materno^c^							
Adequado	42 (4,8)	181 (4,5)	18 (3,3)	37 (3,9)	103 (1,0)	79 (2,9)	< 0,001
Inadequado	693 (79,8)	1 839 (45,5)	281 (51,6)	469 (49,4)	4671 (47,6)	1 277 (44,7)	
Não realizado/ignorado	134 (15,4)	2 025 (50,0)	246 (45,1)	443 (46,7)	5 046 (51,4)	1 439 (52,4)	
Tratamento do parceiro							
Sim	166 (19,1)	546 (13,5)	78 (14,3)	116 (12,2)	772 (7,9)	302 (11,0)	< 0,001
Não	605 (69,6)	2 827 (69,9)	391 (71,8)	667 (70,3)	5 783 (58,9)	1 508 (54,9)	
Ignorado	98 (11,3)	672 (16,6)	76 (13,9)	166 (17,5)	3 265 (33,2)	935 (34,1)	
Desfecho da gestação							
Sífilis congênita recente	806 (92,8)	3 363 (83,1)	507 (93,0)	824 (86,8)	8 856 (90,2)	2 584 (94,1)	< 0,001
Sífilis congênita tardia	15 (1,7)	14 (0,4)	0 (0,0)	5 (0,5)	18 (0,2)	6 (0,2)	
Aborto	19 (2,2)	227 (5,6)	20 (3,7)	33 (3,5)	408 (4,1)	65 (2,4)	
Natimorto	29 (3,3)	441 (10,9)	18 (3,3)	87 (9,2)	538 (5,5)	90 (3,3)	

***Fonte:*** SINAN.

a Exceto idade.

b Teste de homogeneidade de proporções pelo qui-quadrado de Pearson.

c Tratamento adequado para a fase clínica da doença de acordo com o Manual do Ministério da Saúde (10).

Novamente, o Amazonas e o Rio de Janeiro, mas também o Ceará, apresentaram maior proporção de casos em residentes na capital, enquanto que o Espírito Santo e o Rio Grande do Sul distribuíram mais os casos entre outros municípios.

A maior parte das mulheres com sífilis realizou pré-natal, variando entre 67,3% nas amazonenses a 83,3% nas residentes no Distrito Federal. Cerca de 50% das mulheres do Distrito Federal, Espírito Santo e Rio Grande do Sul tiveram o diagnóstico de sífilis durante o pré-natal, com o Amazonas apresentando a menor proporção (31,5%) nessa categoria. No Amazonas, Ceará e Rio de Janeiro, a maioria das mulheres recebeu o diagnóstico na internação para o parto ou curetagem. Mais de 90% delas apresentaram exame não treponêmico reagente no parto/curetagem. A realização de teste treponêmico no parto variou de 19,3% no Ceará a 44,2% no Distrito Federal. O tratamento materno foi considerado inadequado em 44,7% das mulheres no Rio Grande do Sul e em 79,8% das mulheres no Amazonas, com as outras unidades federativas dentro desse intervalo. Os parceiros foram tratados junto com as gestantes em 7,9% dos casos no Rio de Janeiro e 19,1% no Amazonas. Quanto ao desfecho da gestação, no Ceará e no Distrito Federal 83,1% e 93,0% dos casos foram considerados como sífilis congênita, respectivamente. O desfecho de aborto variou de 2,2% no Amazonas até 5,6% no Ceará, e o de natimorto variou de 3,3% no Amazonas, Distrito Federal e Rio Grande do Sul até 10,9% no Ceará.

Na tabela 3 encontram-se as características dos casos de sífilis congênita recente do período. A reatividade ao teste não treponêmico no sangue periférico variou de 61,3% no Amazonas a 72,5% no Ceará. Já a realização de VDRL no líquor foi heterogênea, variando de 5,4% no Amazonas a 62,6% no Rio Grande do Sul, sempre com baixa reatividade, bem como pequena proporção de alterações liquóricas. A radiografia de ossos longos teve maior percentual de realização, entre 19,8% no Amazonas e 64,5% no Distrito Federal. Entre 60 e 78% dos casos de sífilis congênita recente receberam tratamento com penicilina cristalina. Entretanto, entre 7,8% dos casos no Espírito Santo e 19,8% no Rio de Janeiro não foram tratados ou não tinham essa informação disponível. Das 1 042 crianças com sífilis congênita recente e exame de líquor positivo, apenas 77,5% foram tratadas com penicilina cristalina. Entre as que não realizaram o exame de líquor, somente 59,1% receberam esse tratamento.

### Dados do relacionamento das bases de sífilis em gestantes e sífilis congênita

O relacionamento entre as duas bases de dados utilizando a variável nome da gestante e, como variáveis de ligação, nome da mulher com desfecho de sífilis congênita e município de residência, identificou 7 934 pares (43,3%), resultantes de 18 310 notificações de sífilis em gestantes e 18 973 notificações de sífilis congênita. Do total de pares, 4 961 (62,5%) eram residentes no Rio de Janeiro, com uma distribuição mais equilibrada ente os outros estados. A proporção de casos de sífilis em gestantes com desfecho de sífilis congênita foi de 39,1% no Amazonas, 27,4% no Ceará, 19,3% no Distrito Federal, 30,8% no Espírito Santo, 56,8% no Rio de Janeiro e 32,4% no Rio Grande do Sul. Dos casos de sífilis gestacional nos pares de casos aqui formados, 74% tiveram diagnóstico de sífilis durante o pré-natal e 18% no parto. Em 8% dos casos ignorava-se o momento do diagnóstico materno.

**TABELA 3.** Características clínicas, laboratoriais e desfecho dos casos notificadas por sífilis congênita precoce em seis unidades federativas no Brasil, 2007 a 2012

Característica n (%)	Unidade federativa						
	Amazonas No. = 806	Ceará No. = 3 363	Distrito Federal No. = 507	Espírito Santo No. = 824	Rio de Janeiro No. = 8 856	Rio Grande do Sul No. = 2 584	*P*-valor^a^
VDRL sangue periférico							
Reagente	494 (61,3)	2 437 (72,5)	335 (66,1)	540 (65,5)	6 215 (70,2)	1 865 (72,2)	< 0,001
Não reagente	143 (17,7)	546 (16,2)	119 (23,5)	210 (25,5)	1 521 (17,2)	493 (19,1)	
Não realizado/ignorado	169 (21,0)	380 (11,3)	53 (10,4)	74 (9,0)	1 120 (12,6)	226 (8,7)	
VDRL liquor							
Reagente	14 (1,8)	103 (3,1)	13 (2,6)	15 (1,8)	160 (1,8)	99 (3,8)	< 0,001
Não reagente	30 (3,7)	1 178 (35,0)	211 (41,6)	478 (58,0)	3 495 (39,4)	1 519 (58,8)	
Não realizado/ignorado	762 (94,5)	2 082 (61,9)	283 (55,8)	331 (40,2)	5 201 (58,8)	966 (37,4)	
Alteração no liquor							
Sim	11 (1,3)	131 (3,9)	16 (3,1)	48 5,8)	322 (3,6)	230 (8,9)	< 0,001
Não	123 (15,3)	1 120 (33,3)	225 (44,4)	433 (52,5)	2 698 (30,5)	1 355 (52,4)	
Não realizado/ignorado	672 (83,4)	2 112 (62,8)	266 (52,5)	343 (41,6)	5 836 (65,9)	999 (38,7)	
Alteração óssea							
Sim	38 (4,7)	60 (1,7)	15 (3,0)	22 (2,7)	189 (2,1)	97 (3,8)	< 0,001
Não	130 (16,1)	1 337 (39,8)	312 (61,5)	497 (60,3)	4 113 (46,5)	1 524 (59,0)	
Não realizado/ignorado	638 (79,2)	1 966 (58,5)	180 (35,5)	305 (37,0)	4 554 (51,4)	963 (37,2)	
Tratamento do recém-nascido							
Penicilina cristalina	588 (73,0)	2 620 (77,9)	305 (60,2)	569 (69,0)	5 706 (64,4)	1 528 (59,1)	< 0,001
Penicilina procaina	36 (4,5)	137 (4,1)	15 (3,0)	53 (6,4)	541 (6,1)	74 (2,9)	
Penicilina benzatina	10 (1,2)	55 (1,6)	53 (10,4)	60 (7,3)	402 (4,5)	301 (11,7)	
Outro^b^	40 (4,9)	148 (4,4)	82 (16,2)	78 (9,5)	465 (5,2)	234 (9,0)	
Não realizado/ignorado	132 (16,4)	403 (12,0)	52 (10,2)	64 (7,8)	1 742 (19,8)	447 (17,3)	

***Fonte.*** SINAN.

a Teste de homogeneidade de proporções pelo qui-quadrado de Pearson.

b Outro. qualquer tratamento utilizando outro antibiótico que não a penicilina.

Quanto à situação de tratamento materno, das gestantes que tiveram o diagnóstico no pré-natal, o tratamento foi considerado adequado em 266 (4,5%), inadequado em 3 474 (59,2%) e 1 473 (25,2%) não foram tratadas. Em 652 (11,1%) mulheres, essa informação era ignorada.

## DISCUSSÃO

Os dados descritos neste estudo apresentam grande heterogeneidade, refletindo as dimensões continentais e o desenvolvimento contrastante do Brasil. Nos estados do Ceará, Espírito Santo e Rio Grande do Sul, menos de um terço das gestantes com sífilis residia na capital, ao contrário de Amazonas e Rio de Janeiro. Entretanto, no Ceará, 66,4% dos casos de sífilis congênita foram notificados na capital, enquanto que nos outros estados as proporções de sífilis congênita seguiram a tendência da sífilis na gestação. A concentração de casos nas capitais de algumas unidades federativas pode ser atribuída às redes de saúde mais estruturadas ou à presença de profissionais de saúde mais sensibilizados para diagnóstico e notificação dos casos.

Tanto a taxa de detecção de sífilis em gestantes como a taxa de incidência de sífilis congênita apresentaram, neste estudo, uma tendência de aumento, só revertida no Distrito Federal para a sífilis em gestantes (redução de 5,2%) e no Amazonas para a sífilis congênita (0,7%) no período do estudo. Esta tendência de aumento se repete no país como um todo (4), apesar do compromisso de eliminação (12). Recentemente, em um estudo de base hospitalar, Domingues et al. (13) relataram uma prevalência de 1,02% de sífilis na gestação no Brasil. De maneira global, existe uma ressurgência da sífilis adquirida em países desenvolvidos (14). No Brasil, ela é mais concentrada em homens que fazem sexo com homens; mas, certamente, existe a capacidade de transmissão para mulheres em idade reprodutiva e com probabilidade de engravidar (15).

O rastreio de sífilis na gestação é uma das atividades mais custo-efetivas em saúde pública (16). Em países com alta prevalência da infecção, os benefícios são inquestionáveis (17). No Brasil, os municípios devem realizar a triagem pré-natal de acordo com as orientações do Ministério da Saúde, que também faz a distribuição de testes rápidos treponêmicos dentro do Programa Rede Cegonha. Entre 2007 e 2012 houve um incremento da utilização do teste rápido nos cinco estados estudados por nós, com redução no Distrito Federal. O teste rápido para sífilis é uma tecnologia importante, pois pode proporcionar o acesso precoce ao diagnóstico, especialmente em locais com dificuldade para a realização de testes não treponêmicos laboratoriais (18).

Apesar dos avanços tecnológicos agilizando o diagnóstico e o tratamento da gestante, o controle da sífilis na gestação continua sendo um desafio para a atenção pré-natal. A infecção se apresenta assintomática na gestação, justificando a necessidade do rastreio sorológico (2). O tratamento deve ser instituído com penicilina benzatina de acordo com a estágio clínico da infecção (19).

Neste estudo, 62,8% e 45,4% das gestantes do Amazonas e do Ceará, respectivamente, foram classificadas como sífilis primária, situação na qual deveria ter sido identificado o cancro, primeira manifestação clínica da doença. Além disso, 24,1% no Distrito Federal e 18% no Ceará foram classificadas na categoria de sífilis terciária. Ambas as situações aqui relatadas estão em desacordo com a literatura, sugerindo desconhecimento sobre a doença ou preenchimento errôneo da ficha de notificação, fonte de dados desta pesquisa. Quando analisado o tratamento instituído na gestante e a fase clínica relatada na notificação, verificou-se que a terapia prescrita estava em desacordo para uma proporção entre 38,1% no Distrito Federal e 54,4% no Rio de Janeiro. De forma semelhante, um estudo realizado no Rio de Janeiro mostrou o despreparo dos profissionais das equipes de saúde em relação ao tratamento da sífilis na gestação (20).

Aproximadamente 67,3% (Amazonas) e 83,3% (Distrito Federal) das mulheres com desfecho de sífilis congênita realizaram pré-natal. Entre essas, 31,5% (Amazonas) e 51,6% (Distrito Federal) foram diagnosticadas durante esse período, evidenciando provável falha no diagnóstico e tratamento materno para a prevenção da sífilis congênita, como já relatado em outros estados brasileiros (21, 22). O tratamento dos parceiros tem sido negligenciado nesse grupo, tendo variado, no presente estudo, de 7,9% no Rio de Janeiro a 19,1% no Amazonas.

Na avaliação dos casos de sífilis congênita notificados ao SINAN, verificou-se baixa realização de exame de VDRL no líquor dos recém-nascidos no Amazonas (5,5%), valor bem inferior ao encontrado no Rio Grande do Sul (62,6%). Entretanto, a realização de exame do líquor é mandatória para a decisão de usar penicilina procaína intramuscular ao invés da cristalina intravenosa (5). A proporção de recém-nascidos sem exame de líquor que foi tratada com penicilina cristalina corretamente foi de 59,1%, reforçando a tese do desconhecimento de protocolos clínicos (5).

As perdas fetais relacionadas à sífilis congênita variaram quanto aos abortos de 2,2% no Amazonas a 5,6% no Ceará. Quanto às perdas fetais tardias ou natimortos, a variação foi de 3,3% no Amazonas, Distrito Federal e Rio Grande do Sul a 10,9% no Ceará. Recentemente, a Organização Mundial da Saúde (OMS) estabeleceu como indicador para o progresso dos países em direção à eliminação de sífilis congênita uma meta de manter os natimortos por sífilis abaixo de 2% entre o total de perdas fetais (16), valor que os cinco estados e o Distrito Federal ainda estão longe de alcançar. O relacionamento entre as duas bases de dados do SINAN mostrou que grande parte das mulheres que teve diagnóstico de sífilis na gravidez não logrou ter o caso de sífilis congênita prevenido em seu concepto.

Entre as limitações do presente estudo encontra-se a utilização de dados secundários de notificação, que podem ser considerados somente uma abordagem aproximada do problema da sífilis. Entretanto, ao se levar em consideração que a sífilis congênita é uma doença de notificação compulsória desde 1986 (7), pode-se acreditar que os dados coletados tracem um perfil, ainda que não representem a totalidade dos casos. Outra limitação foi o grande número de informações ignoradas nas bases de dados oficiais, o que também dificulta o estabelecimento do perfil epidemiológico da sífilis em gestantes e de sífilis congênita no Brasil, prejudicando o planejamento de ações eficazes de eliminação no país. Esse mesmo quadro se repete em outros países da América Latina e Caribe, sendo importante enfatizar a necessidade de melhorar a capacidade dos países de coletarem dados de alta qualidade na cobertura de intervenções e desigualdades e utilizarem esses dados como base de decisões para a melhora da atenção a gestantes e crianças (23). Melhores indicadores servirão também para que o país possa monitorar adequadamente seu status em relação às metas de eliminação da sífilis congênita.

## CONCLUSÕES

Os resultados deste estudo mostraram, para o período do estudo, um incremento nas taxas de detecção de sífilis. Essas taxas podem ter sido impulsionadas pelo aumento na notificação dos casos. A melhor organização dos serviços de saúde e sensibilização dos profissionais podem diminuir as falhas na prevenção e assistência aos casos de sífilis. O monitoramento constante dos casos de sífilis em gestantes e de sífilis congênita por meio do sistema de vigilância é essencial para que o Brasil se encaminhe para o cumprimento dos objetivos de eliminação da sífilis congênita estabelecidos pela Organização Pan-Americana da Saúde (OPAS) e pela OMS.

### Agradecimentos.

O estudo recebeu apoio financeiro do Ministério da Saúde, Secretaria Executiva, Fundação Nacional de Saúde, Brasil, mediante termo de Cooperação nº 323/2013 - Processo nº 25000.202637/2013-92.

### Declaração de responsabilidade.

A responsabilidade pelas opiniões expressas neste manuscrito é estritamente dos autores e não reflete necessariamente as opiniões ou políticas da *RPSP/PAJPH* nem da OPAS.

## References

[1] 1. Rothschild BM. The history of syphilis. Clin Infect Dis. 2005;40:1454–63. doi: 10.1086/429626 10.1086/429626 15844068

[2] 2. Goldenberg RL, Culhane JF, Johnson DC. Maternal infection and adverse fetal and neonatal outcomes. Clin Perinatol. 2005;32(3): 523–59. doi: 10.1016/j.clp.2005.04.006. 10.1016/j.clp.2005.04.006 PMC7119141 16085019

[3] 3. Newman L, Kamb M, Hawkes S, Gomez G, Say L, Seuc A, et al. Global estimates of syphilis in pregnancy and associated adverse outcomes: Analysis of multinational antenatal surveillance data. PLOS Medicine. 2013;10(2). doi: 10.1371/journal.pmed.1001396. 10.1371/journal.pmed.1001396 PMC3582608 23468598

[4] 4. Brasil, Ministério da Saúde, Secretaria de Vigilância em Saúde, Departamento de DST, Aids e Hepatites Virais. Boletim Epidemiológico - Sífilis. Ano IV, no 1. Brasília, 2015. Disponível em: http://www.aids.gov.br/sites/default/files/anexos/publicacao/2015/57978/_p_boletim_sifilis_2015_fechado_pdf_p__18327.pdf Acessado em agosto de 2016.

[5] 5. Schmid G, Stoner BP, Hawkes S, Broutet N. The need and plan for global elimination of congenital syphilis. Sex Transm Dis. 2007;34:S5–S10. doi: 10.1097/01.olq.0000261456.09797.1b. 10.1097/01.olq.0000261456.09797.1b 17592390

[6] 6. Follett T1, Clarke DF. Resurgence of congenital syphilis: diagnosis and treatment. Neonatal Netw. 2011;30(5):320–8. doi: 10.1891/0730-0832.30.5.320. 10.1891/0730-0832.30.5.320 21846627

[7] 7. Brasil, Ministério da Saúde. Portaria 542/1986. Diário Oficial da União, 24 dez 1986, Seção 1, p. 19827. Disponível em: http://pesquisa.bvsalud.org/ses/resource/pt/crt-3619 Acessado em janeiro de 2017.

[8] 8. Brasil, Ministério da Saúde, Secretaria de Vigilância em Saúde. Portaria 5/2006. Diário Oficial da União, 22 fev 2006, Seção 1, p. 34. Disponível em: http://redsang.ial.sp.gov.br/site/docs_leis/ag/ag6.pdf Acessado em janeiro de 2017.

[9] 9. Saraceni V, Miranda AE. Coverage by the Family Health Strategy and diagnosis of syphilis in pregnancy and congenital syphilis. Cad Saude Publica. 2012;28(3):490–6. 10.1590/s0102-311x2012000300009 22415181

[10] 10. Brasil, Ministério da Saúde, Secretaria de Vigilância em Saúde, Programa Nacional de DST/AIDS. Diretrizes para o controle da sífilis congênita: manual de bolso. 2a ed. Brasília: Ministério da Saúde; 2006. Disponível em: http://www.aids.gov.br/publicacao/2006/diretrizes-para-o-controle-da-sifilis-congenita-manual-de-bolso Acessado em janeiro de 2017.

[11] 11. Camargo Jr. KR, Coeli CM. Reclink: aplicativo para o relacionamento de bases de dados, implementando o método probabilistic record linkage. Cad Saude Publica. 2000;16(2):439–47. doi: 10.1590/S0102-311X2012000300009 10883042

[12] 12. Brasil, Ministério da Saúde. Transmissão vertical do HIV e sífilis: estratégias para redução e eliminação. Brasília: Ministério da Saúde; 2014. Disponível em: http://www.aids.gov.br/publicacao/2014/transmissao-vertical-do-hiv-e-sifilis-estrategias-para-reducao-e-eliminacao Acessado em janeiro de 2017.

[13] 13. Domingues RM, Szwarcwald CL, Souza Junior PR, Leal Mdo C. Prevalence of syphilis in pregnancy and prenatal syphilis testing in Brazil: birth in Brazil study. Rev Saude Publica. 2014;48(5):766–74. 10.1590/S0034-8910.2014048005114 PMC4211581 25372167

[14] 14. Read P, Fairley CK, Chow EP. Increasing trends of syphilis among men who have sex with men in high income countries. Sex Health. 2015 Apr;12(2):155–63. doi: 10.1071/SH14153. 10.1071/SH14153 25607751

[15] 15. Fernandes FR, Zanini PB, Rezende GR, Castro LS, Bandeira LM, Puga MA, et al. Syphilis infection, sexual practices and bisexual behaviour among men who have sex with men and transgender women: a cross-sectional study. Sex Transm Infect. 2015;91(2):142-9. doi: 10.1136/sex-trans-2014-051589. Epub 2014 Oct 9. 10.1136/sextrans-2014-051589 25301711

[16] 16. Kamb ML, Newman LM, Riley PL, Mark J, Hawkes SJ, Malik T, et al. A road map for the global elimination of congenital syphilis. Obstet Gynecol Int. 2010; 2010. pii: 312798. doi: 10.1155/2010/312798. Epub 2010 Jul 14. 10.1155/2010/312798 PMC2913802 20706693

[17] 17. Kahn JG, Jiwani A, Gomez GB, Hawkes SJ, Chesson HW, Broutet N, et al. The cost and cost-effectiveness of scaling up screening and treatment of syphilis in pregnancy: a model. PLoS One. 2014;9(1):e87510. doi: 10.1371/journal.pone.0087510. eCollection 2014. 10.1371/journal.pone.0087510 PMC3906198 24489931

[18] 18. Swartzendruber A, Steiner RJ, Adler MR, Kamb ML, Newman LM. Introduction of rapid syphilis testing in antenatal care: A systematic review of the impact on HIV and syphilis testing uptake and coverage. Int J Gynaecol Obstet. 2015 Apr 29. pii: S0020-7292(15)00205-2. doi: 10.1016/j. ijgo.2015.04.008. 10.1016/j.ijgo.2015.04.008 PMC6799988 26001704

[19] 19. Clement ME, Okeke NL, Hicks CB. Treatment of syphilis: a systematic review. JAMA. 2014;312(18):1905–17. doi: 10.1001/jama.2014.13259 10.1001/jama.2014.13259 PMC6690208 25387188

[20] 20. Domingues RM, Lauria Lde M, Saraceni V, Leal Mdo C. Manejo da sífilis na gestação: conhecimentos, práticas e atitudes dos profissionais pré-natalistas da rede SUS do município do Rio de Janeiro. Cienc Saude Colet. 2013;18(5):1341–51. doi: 10.1590/S1413-81232013000500019 23670462

[21] 21. Oliveira LR, Costa Mda C, Barreto FR, Pereira SM, Dourado I, Teixeira MG. Evaluation of preventative and control measures for congenital syphilis in State of Mato Grosso. Rev Soc Bras Med Trop. 2014;47(3):334–40. 10.1590/0037-8682-0030-2014 25075485

[22] 22. Serafim AS, Moretti GP, Serafim GS, Niero CV, da Rosa MI, Pires MM, et al. Incidence of congenital syphilis in the South Region of Brazil. Rev Soc Bras Med Trop. 2014;47(2):170–8. doi: 10.1590/0037-8682-0030-2014. 24861290

[23] 23. Serruya SJ, Duran P, Martinez G, Romero M, Caffe S, Alonso M, et al. Maternal and congenital syphilis in selected Latin America and Caribbean countries: a multi-country analysis using data from the Perinatal Information System. Sex Health. 2015;12(2):164–9. doi: 10.1071/SH14191. 10.1071/SH14191 25725563

